# Improving CSF Biomarkers’ Performance for Predicting Progression from Mild Cognitive Impairment to Alzheimer’s Disease by Considering Different Confounding Factors: A Meta-Analysis

**DOI:** 10.3389/fnagi.2014.00287

**Published:** 2014-10-16

**Authors:** Daniel Ferreira, Amado Rivero-Santana, Lilisbeth Perestelo-Pérez, Eric Westman, Lars-Olof Wahlund, Antonio Sarría, Pedro Serrano-Aguilar

**Affiliations:** ^1^Division of Clinical Geriatrics, Department of Neurobiology, Care Sciences and Society, Karolinska Institutet, Stockholm, Sweden; ^2^Canarian Foundation of Health and Research (FUNCIS), Las Palmas de Gran Canaria, Spain; ^3^Red de Investigación en Servicios de Salud en Enfermedades Crónicas (REDISSEC), Santa Cruz de Tenerife, Spain; ^4^Evaluation Unit of the Canary Islands Health Service (SESCS), Santa Cruz de Tenerife, Spain; ^5^Center for Biomedical Research of the Canary Islands (CIBICAN), University of La Laguna, Tenerife, Spain; ^6^Agency for Health Technology Assessment (AETS), Institute of Health Carlos III, Madrid, Spain

**Keywords:** mild cognitive impairment, Alzheimer’s disease, CSF biomarkers, confounding factors, meta-analysis, systematic review

## Abstract

**Background:** Cerebrospinal fluid (CSF) biomarkers’ performance for predicting conversion from mild cognitive impairment (MCI) to Alzheimer’s disease (AD) is still suboptimal.

**Objective:** By considering several confounding factors we aimed to identify in which situations these CSF biomarkers can be useful.

**Data Sources:** A systematic review was conducted on MEDLINE, PreMedline, EMBASE, PsycInfo, CINAHL, Cochrane, and CRD (1990–2013).

**Eligibility Criteria:** (1) Prospective studies of CSF biomarkers’ performance for predicting conversion from MCI to AD/dementia; (2) inclusion of Aβ42 and T-tau and/or p-tau. Several meta-analyses were performed.

**Results:** Aβ42/p-tau ratio had high capacity to predict conversion to AD in MCI patients younger than 70 years. The p-tau had high capacity to identify MCI cases converting to AD in ≤24 months.

**Conclusions:** Explaining how different confounding factors influence CSF biomarkers’ predictive performance is mandatory to elaborate a definitive map of situations, where these CSF biomarkers are useful both in clinics and research.

## Introduction

Mild cognitive impairment (MCI) is a high risk factor for developing dementia, particularly Alzheimer’s disease (AD). About 35% of MCI patients progress to AD, with an annual conversion rate of 5–10% (Mitchell, 2009). Because AD entails severe consequences, an appropriate prediction of MCI outcome is crucial for giving the patients a prognosis and to initiate therapeutical strategies as soon as possible. In this regard, the new MCI diagnostic criteria recommended by the National Institute of Aging – Alzheimer’s Association (NIA-AA) emphasize the use of neuroimaging and cerebrospinal fluid (CSF) biomarkers (Albert et al., 2011). Although significant advances have been made in the field of neuroimaging, biomarkers based on CSF are at present the most convenient for studying disease progression.

The currently validated CSF biomarkers of AD are Aβ_42_, total tau (T-tau), and phosphorylated tau (p-tau). CSF Aβ_42_ is reduced, and T-tau and p-tau levels are increased in MCI patients compared to healthy controls (Diniz et al., 2008). In addition, MCI patients with abnormal CSF biomarkers have increased risk to progress to AD (Herukka et al., 2005; Hansson et al., 2006, 2007; Bouwman et al., 2007; Brys et al., 2009; Mattsson et al., 2009; Shaw et al., 2009; Hertze et al., 2010; Buchhave et al., 2012). Buchhave et al. (2012) showed that 90% of MCI patients with pathologic CSF biomarkers developed AD within 9⋅2 years. This knowledge is now incorporated in the new diagnostic criteria for MCI, indicating that positive biomarkers of Aβ accumulation (e.g., CSF Aβ_42_) and neuronal injury (e.g., CSF T-tau and p-tau) confers the highest likelihood that AD pathophysiological processes are the cause of the cognitive dysfunction; and that individuals with this biomarker profile are more likely to decline or progress to dementia due to AD in relatively short periods (Albert et al., 2011). Regarding predictive capacity, although single CSF biomarkers have shown unsatisfactory results, their combination could be suitable to identify which MCI patients will progress to dementia (Ferreira et al., 2014). In particular, the Aβ_42_/p-tau ratio has demonstrated high efficiency (Hansson et al., 2006; Mattsson et al., 2009; Buchhave et al., 2012; Parnetti et al., 2012; Roe et al., 2013). Two systematic reviews with meta-analysis have previously been published (Mitchell, 2009; Monge-Argilés et al., 2010). Mitchell (2009) only evaluated p-tau. Monge-Argilés et al. (2010) evaluated the three CSF biomarkers, but the group of MCI patients that converted to AD was compared to a mixed group of stable MCI cases and MCI patients that converted to non-AD dementias. Moreover, their analysis of combined CSF biomarkers was limited to only three studies and the combination procedure was not sufficiently detailed.

Importantly, CSF biomarkers’ predictive performance could be improved by considering different confounding factors such as the MCI subtype, time to AD conversion, and age (Ferreira et al., 2014). Previous studies show that the CSF biomarkers have better predictive capacity in amnestic MCI (Vos et al., 2013), MCI patients that convert to AD in relatively short periods (e.g., <12 months) (Gaser et al., 2013), and young MCI patients (Mattsson et al., 2012). However, most of the studies performed to date do not consider these confounding factors. These aspects together with methodological variability have made it difficult to propose definitive cut-off values for CSF biomarkers. For this reason, the fact of disseminating the use of CSF biomarkers to clinical routine is compromised at present (Ferreira et al., 2014).

The main objective in this study was to carefully evaluate the capacity of the CSF biomarkers to predict conversion from MCI to AD in several clinically relevant situations. In particular, we aimed to identify for which specific MCI patients these CSF biomarkers might be useful in clinical practice. In order to address this question, several meta-analyses were performed for studies that prospectively analyzed the predictive performance of CSF Aβ_42_ and T-tau and/or p-tau. The design of the included studies is baseline cross-sectional comparisons between MCI patients that convert to AD or dementia (MCI-C) and MCI patients that remain stable (MCI-S) at follow-up. We hypothesized that combined CSF biomarkers would have better predictive performance than single CSF biomarkers, and that this performance could be increased by controlling for different confounding factors such as the MCI subtype, time to AD conversion, and age, among others.

## Material and Methods

### Search strategy and selection criteria

A systematic review was conducted for the period between January 1990 and September 2013 in the following electronic databases: MEDLINE and PreMedline, EMBASE, PsycInfo, CINAHL, Cochrane Library, and CRD. The search strategy was developed for each database using the following Medical Subject Heading (MeSH) and free-text terms: “Alzheimer’s disease diagnosis” or “Alzheimer’s disease,” and “abeta-42” or “T-tau” or “P-tau” or “tau” or “phospho-tau” or “phosphorylated tau.” Examples for the two major databases are shown in Table S1 in Supplementary Materials (MEDLINE) and Table S2 in Supplementary Materials (EMBASE). In addition, reference sections were searched to identify relevant publications.

**Figure 1:**
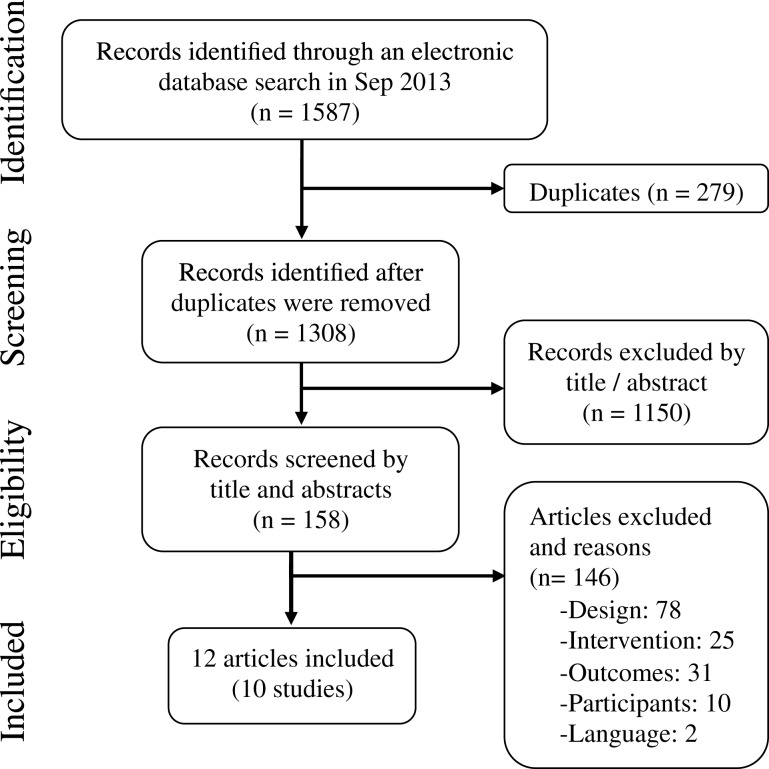
**Study selection flow**.

Inclusion criteria for this meta-analysis were studies that (1) performed a prospective analysis of the CSF biomarkers’ performance for predicting conversion to AD or dementia in individuals with MCI at baseline; (2) included at least two CSF biomarkers, being Aβ_42_ always required along with T-tau and/or p-tau; and (3) were published in English or Spanish. Studies were excluded if they did not report sensitivity or specificity values, or any other data that enabled its calculation. Two reviewers independently performed the study selection (Daniel Ferreira and Amado Rivero-Santana), and in case of doubt and/or disagreements a third reviewer was consulted (Lilisbeth Perestelo-Pérez). The search yielded 1308 references after discarding duplicates. One-hundred fifty-eight articles were selected by title and abstract. After applying eligibility criteria, 12 articles were eventually included (Hampel et al., 2004; Herukka et al., 2005; Parnetti et al., 2006, 2012; Eckerström et al., 2010; Hertze et al., 2010; Monge-Argilés et al., 2011; Buchhave et al., 2012; Ewers et al., 2012; Gaser et al., 2013; Toledo et al., 2013; Vos et al., 2013). Three of these studies included data from the Alzheimer’s Disease Neuroimaging Initiative (ADNI). As these studies represent different analyses of overlapping ADNI subsamples, only one ADNI study was included for each meta-analysis depending on the analyzed biomarker. If two ADNI studies were available for the same biomarker, the one with largest sample was selected. Selection flow including reasons for study exclusion at each phase is shown in Figure 1.

### Data collection, risk of bias, and evaluation of methodological quality

A data extraction sheet was developed to collect relevant data by covering: author and publication year, country, objectives, methods (with special attention to participants’ recruitment procedures, study design, follow-up length, CSF biomarkers evaluated including Aβ_42_/T-tau and Aβ_42_/p-tau ratios, diagnostic groups characteristics, and statistical analyses), results, and conclusions. Data extraction was carried out by two researchers (Daniel Ferreira and Amado Rivero-Santana), and quality and accuracy of the extraction was verified by a third researcher (Lilisbeth Perestelo-Pérez).

Several strategies were followed in order to reduce the risk of bias related to publication, data availability, and reviewer selection (see Table S3 in Supplementary Materials). The QUADAS-2 scale (Whiting et al., 2011) was used in order to assess the methodological quality of the included studies. The scale was applied by two researchers (Amado Rivero-Santana and Daniel Ferreira), and in case of doubt and/or disagreements a third was consulted (Lilisbeth Perestelo-Pérez). Finally, this study was performed in accordance with the PRISMA statement (Liberati et al., 2009; Moher et al., 2010), which provides a detailed guideline of preferred reporting style for systematic reviews and meta-analyses.

### Identification of potential confounding factors of CSF biomarkers’ predictive performance

We hypothesized that CSF biomarkers’ predictive performance might be influenced by different confounding factors. To explore sources of heterogeneity, the following factors were defined *a priori* based on the literature (see Table 1 for a detailed description of the different factors and considered categories): (1) recruitment setting; (2) MCI subtype; (3) diagnostic criteria for MCI at baseline; (4) diagnostic criteria for AD at follow-up; (5) postmortem confirmation of AD pathology; (6) criteria for conversion from MCI to AD/dementia; (7) diagnosis at follow-up; (8) follow-up length (as rough estimation of time to AD conversion); (9–11) MCI severity at baseline according to mini-mental state examination (MMSE), clinical rating [e.g., clinical dementia rating (CDR), global deterioration scale (GDS)], and magnetic resonance imaging (MRI) rating (i.e., degree of brain atrophy); (12) Age; (13) gender distribution; (14) years of education; (15) family history of AD; (16) APOE e4 status; (17) technology applied for the CSF analysis; and (18) cut-offs for interpreting the CSF levels.

**Table 1:** **Potential confounding factors of CSF biomarkers’ predictive performance**.

Factors	Considered categories
**RECRUITMENT**	
Setting	Specialized centers (9 + 1) vs. primary care (0) vs. general population (0)
**DIAGNOSTIC ISSUES**	
MCI subtype	Amnestic MCI (6 + 1) vs. non-amnestic MCI (1) vs. mixed sample (2)^a^ vs. non-specified (1)
Diagnostic criteria for MCI at baseline	Petersen et al. (1999, 2001); Petersen, 2004 or Petersen’s group 2006^b^ (8) vs. Winblad et al. (2004) (1) vs. ADNI criteria (+ 1)
Diagnostic criteria for AD at follow-up	NINCDS-ADRDA (8 + 1) vs. non-specified (1)
Postmortem confirmation of AD pathology	All the studies lacked postmortem confirmation of AD pathology (0)
Diagnosis at follow-up	AD (6 + 1) vs. mixed group of dementias (3)^c^
Criteria for conversion	Fulfillment of diagnostic criteria (7 + 1) vs. Decline in clinical scales (e.g., CDR) (1) vs. non-specified (1)
**TIME TO AD CONVERSION**	
Time to AD conversion	Follow-up ≤24 months (5 + 1) vs. Follow-up >24 months (4)
**MCI SEVERITY AT BASELINE**	
MMSE total score	No enough variability: all studies reporting MMSE have mean scores between 23 and 30 (7 + 1) vs. non-specified (2)
Clinical rating	CDR = 0.5/GDS = 3 (5 + 1) vs. Non-specified (4)
MRI rating	Only available for 2 (+ 1) studies, with variability in procedures
**DEMOGRAPHICS/RISK FACTORS AT BASELINE**	
Age	≤70 years (4) vs. >70 years (4 + 1) vs. non-specified (1)
Gender	Results are never presented separately for men and women (0)
Education	≤12 years (3) vs. >12 years (+ 1) vs. non-specified (6)
Family history of AD	Not enough information (1)
APOE e4 status	Not enough information (3 + 1)
**CSF METHODS**	
Technology for CSF analysis	ELISA (6) vs. xMAP (3 + 1)
Cut-offs for interpreting CSF levels	Great variability: Internal (highest value of SN + SP or Youden’s Index) (3) vs. External or independent of clinical diagnosis (Mixture model analysis, obtained from another cohort in the same study; Hulstaert et al., 1999; Sjögren et al., 2001; Shaw et al., 2009; Zetterberg et al., 2003) (4 + 1) vs. both internal and external (2)

*Between brackets, number of studies available for at least one biomarker; +1 refers to ADNI studies (only one ADNI study is included for each subgroup meta-analysis)*.

*^a^Herukka et al. (2005) included amnestic MCI patients as well as patients with other types of cognitive decline. Monge-Argilés et al. (2011) included a mixed group of amnestic MCI and non-amnestic MCI patients*.

*^b^Diagnostic criteria for MCI published by Petersen’s group in Artero et al. (2006)*.

*^c^Both in Parnetti et al. (2006) and Monge-Argilés et al. (2011) it is unclear whether MCI converters progressed exclusively to AD*.

*MCI, mild cognitive impairment; AD, Alzheimer’s disease; MMSE, mini-mental state examination; MRI, magnetic resonance imaging; APOE, apolipoprotein E; CSF, cerebrospinal fluid; ADNI: Alzheimer’s disease neuroimaging initiative; NINCDS-ADRDA, National Institute of Neurological and Communicative Disorders and Stroke and the Alzheimer’s Disease and Related Disorders Association; CDR, clinical dementia rating; GDS, global deterioration scale*.

### Statistical analysis

**Figure 2:**
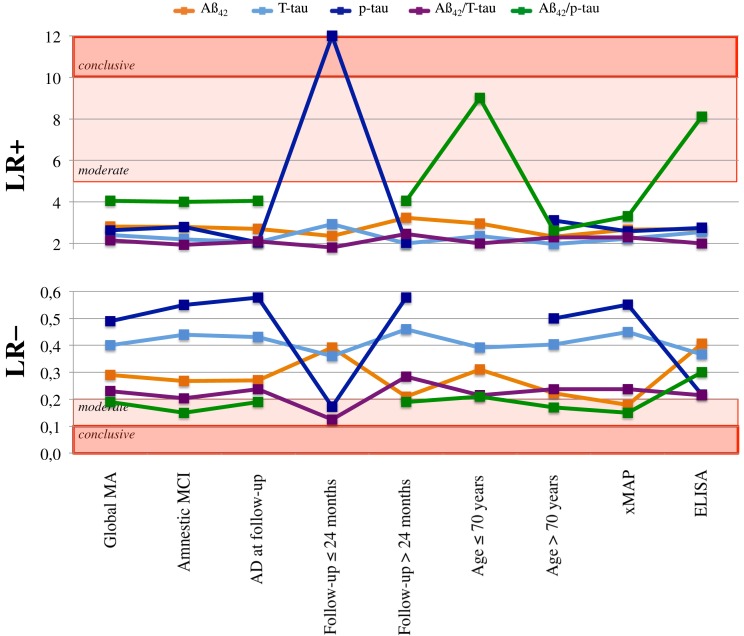
**Positive and negative likelihood ratios**. A LR+ greater than one increases the pretest probability that the disease is present [in this context progression from MCI to AD or, in other words, MCI due to AD (Albert et al., 2011)]. A LR– of less than one diminishes the pretest probability that disease is present. The established guidelines (Qizilbash, 2002) states that a LR+ greater than 10 will often make conclusive changes to the pretest probability, indicating that the disease is likely present; a LR+ between 5 and 10 corresponds to moderate increase in probability; and a LR+ between 2 and 5 corresponds to small increase. A LR− of less than 0⋅1 will often make conclusive changes to the pretest probability that the disease is present, indicating that the disease is unlikely present; a LR− between 0⋅1 and 0⋅2 corresponds to moderate decrease in probability; and a LR− between 0⋅2 and 0⋅5 corresponds to small decrease. LR+, positive likelihood ratio; LR−, negative likelihood ratio; Global MA, global meta-analysis; MCI, mild cognitive impairment; AD, Alzheimer’s disease.

For each article, true and false positives/negatives values were calculated from sensitivity, specificity, positive predicted value, negative predicted value, and/or the rate of converters and non-converters. A global meta-analysis was performed for each single CSF biomarker (i.e., Aβ_42_, T-tau, and p-tau) and two relevant ratios (i.e., Aβ_42_/T-tau and Aβ_42_/p-tau). Analyses were performed with the MetaDisc 1.1.1 software (Zamora et al., 2006). Sensitivity and specificity pooled estimates were calculated with random-effects models (DerSimonian and Laird, 1986), which yield more conservative estimates. For a qualitative interpretation of sensitivity and specificity results, values above 80% were considered indicative of satisfactory predictive performance according to international recommendations (The Ronald and Nancy Reagan Research Institute of the Alzheimer’s Association and the national Institute on Aging working Group, 1998). Positive and negative likelihood ratios were calculated from resulting sensitivity and specificity values and interpreted following established guidelines (see these guidelines in Figure 2 footnotes) (Qizilbash, 2002). Likelihood ratios indicate how the pretest probability of disease is increased or decreased by the outcome of a diagnostic test. A positive likelihood ratio [LR + = sensitivity/(1 – specificity)] greater than one increases the probability that the disease is present (in this context progression to AD) and helps to rule-in MCI-C cases. A negative likelihood ratio (LR– = (1 – sensitivity)/specificity) of less than one diminishes the probability that disease is present and helps to rule-out MCI-C cases. Statistical heterogeneity was explored with the Cochran *Q*-test. As this statistic has low power when few studies are available, a recommended p-value of 0⋅10 was established as statistical significance threshold to detect heterogeneity (Hardy and Thompson, 1998). Differences in sensitivity and specificity values for pairs of subgroup meta-analyses (e.g., MCI cases younger than 70 years vs. older than 70 years) were tested with the formula: *Q*_BET_ = *Q*_TOT_ – (*Q*_1_ + *Q*_2_). Where *Q*_TOT_ represents the overall inter-study variability, and *Q*_1_ and *Q*_2_ represents inter-study variability for each subgroup in the comparison (Deeks et al., 2001). The *Q*_BET_ statistic was then compared to a χ^2^ distribution with *J −* 1 degrees of freedom using a significance level of 0⋅05, where *J* is the number of subgroups.

## Results

### Main characteristics of included studies and methodological quality

Among the 12 studies included, 10 offered data about the diagnostic performance of Aβ_42_, 6 about T-tau, 5 about p-tau, and 6 about the Aβ_42_/T-tau and Aβ_42_/p-tau ratios. Main study characteristics are detailed in Table 2. Methodological quality (QUADAS-2) is shown in Table S4 in Supplementary Materials. In summary, (1) Patient selection: only two studies demonstrated low risk of bias; seven did not explicitly state consecutive or random samples; patients could have been inappropriately excluded in nine studies. (2) Diagnostic test: seven studies proved low risk of bias by using external cut-off values or establishing the cut-off in the study sample independently of the clinical diagnosis [i.e., mixture model analysis in Buchhave et al., 2012]. (3) Diagnostic criterion: all the studies were classified as unclear given that postmortem confirmation of AD pathology was never performed. (4) Patients flow and follow-up: three studies demonstrated low risk of bias; all the studies applied the same reference standard to all the patients, but patients were followed during only two years or less in five studies; six studies did not include all baseline patients in the final analyses.

**Table 2:** **Main characteristics of included studies**.

	Biomarkers^a^	Sample size (*N*)^b^	Comparison	Mean age	Women (%)	MCI subtype	Diagnostic criteria for MCI	Diagnostic criteria for AD	CSF method	Cut-off value	Follow-up (years)
Buchhave et al. (2012)	Aβ_42_, Aβ_42_/p-tau	134	MCI-C_AD_ vs. (MCI-S + MCI-C_other_)	69.8	55	aMCI	Petersen (2004)	NINCDS-ADRDA	xMAP	Mixture model analysis	9.2
Eckerström et al. (2010)	Aβ_42_, Aβ_42_/T-tau	68	MCI-C vs. (MCI-S + controls)	67.8	61.8	n.s.	Winblad et al. (2004)	NINCDS-ADRDA	ELISA	Internal (maximum value of SN + SP)	2
Ewers et al. (2012)	T-tau, p-tau	130	MCI-C_AD_ vs. MCI-S	73.9	63.8	aMCI	ADNI^e^	NINCDS-ADRDA	xMAP	n.s.	2.3
Gaser et al. (2013)	Aβ_42_, Aβ_42_/p-tau	99	MCI-C_AD_ vs. MCI-S	75.3	n.s.	aMCI	ADNI^e^	NINCDS-ADRDA	xMAP	n.s.	3
Hampel et al. (2004)	Aβ_42_, T-tau	52	MCI-C_AD_ vs. MCI-S	72.4	59	aMCI	Petersen et al. (1999)	NINCDS-ADRDA	ELISA	Internal (maximum value of SN + SP)	0.7
Hertze et al. (2010)	Aβ_42_, T-tau, p-tau, Aβ_42_/T-tau Aβ_42_/p-tau	159	MCI-C_AD_ vs. (MCI-S + MCI-C_other_)	71.8	57.2	aMCI	Petersen (2004)	NINCDS-ADRDA	xMAP	AD vs. control in the same study (highest value of Youden’s index)	4.7
Herukka et al. (2005)	Aβ_42_, T-tau, p-tau, Aβ_42_/T-tau, Aβ_42_/p-tau	78	MCI-C_AD_ vs. MCI-S	70.4	59	aMCI + naMCI	Petersen (2004)	n.s.	ELISA	Internal Sjögren et al. (2001); Hulstaert et al. (1999)	3
Monge-Argilés et al. (2011)	Aβ_42_, T-tau, p-tau, Aβ_42_/T-tau, Aβ_42_/p-tau	38	MCI-C vs. controls	73.5	63.2	aMCI + naMCI	Artero et al. (2006)^d^	NINCDS-ADRDA	xMAP	Internal Shaw et al. (2009)	0.5
Parnetti et al. (2006)	Aβ_42_, T-tau, p-tau	44	MCI-C vs. MCI-S	n.s.	n.s.	aMCI	Petersen et al. (1999)	NINCDS-ADRDA	ELISA	Aβ_42_, T-tau: Sjögren et al. (2001), p-tau: Zetterberg et al. (2003)	1
Parnetti et al. (2012)	Aβ_42_, Aβ_42_/p-tau	90	MCI-C_AD_ vs. MCI-S	66.7	48.9	aMCI	Petersen et al. (1999)	NINCDS-ADRDA	ELISA	Internal (highest value of Youden’s index)	3.4
Toledo et al. (2013)	Aβ_42_/T-tau	122	MCI-C_AD_ vs. MCI-S	74	33.2	aMCI	ADNI^e^	NINCDS-ADRDA	xMAP	Shaw et al. (2009)	3.2
Vos et al. (2013)	Aβ_42_, Aβ_42_/T-tau	191	MCI-C_AD_ vs. MCI-S	70.7^c^	53.6^c^	aMCI and naMCI	Petersen (2004)	NINCDS-ADRDA	ELISA	Aβ_42_: Sjögren et al. (2001) Aβ_42_/T-tau: Hulstaert et al. (1999)	2

*^a^Biomarkers with suitable data for performing meta-analyses*.

*^b^Sample size included in the meta-analyses*.

*^c^Results were only available for the whole MCI sample (N = 625)*.

*^d^Artero et al. (2006)*.

*^e^MCI diagnostic criteria in the ADNI cohort are comparable to Petersen et al. (1999)*.

*n.s., non-specified; MCI, mild cognitive impairment; MCI-C, MCI patients that convert to AD or other non-AD dementia form; MCI-C_AD_, MCI-C patients that exclusively convert to AD; MCI-C_other_, MCI-C patients that convert to a non-AD dementia form; MCI-S, MCI patients that remain stable at follow-up; aMCI, amnestic MCI; naMCI, non-amnestic MCI; AD, Alzheimer’s disease; CSF, cerebrospinal fluid; ADNI, Alzheimer’s Disease Neuroimaging Initiative; NINCDS-ADRDA, National Institute of Neurological and Communicative Disorders and Stroke and the Alzheimer’s Disease and Related Disorders Association; SN + SP, sensitivity + specificity*.

### Global meta-analysis: CSF biomarkers’ predictive performance

Table 3 shows sensitivity and specificity values with 95% CI, heterogeneity, and likelihood ratios. Heterogeneity was significant for the three single biomarkers as well as for the two evaluated ratios. Aβ_42_/p-tau ratio showed the best performance with 85% sensitivity, 79% specificity, and a negative likelihood ratio of 0⋅19, indicating moderate decrease in the probability that the disease is present.

**Table 3:** **Global meta-analysis and subgroups meta-analyses**.

Biomarker	Meta-analysis	Sensitivity (%)	*Q*	Inter-groups difference (*Q*_BET)_	Specificity (%)	*Q*	Inter-groups difference (*Q*_BET)_	LR+	LR−
Aβ_42_	Global (*n* = 10)^a^	79 (75–83)	47.88***	–	72 (68–75)	60.19***	–	2.82	0.29
	Amnestic MCI (*n* = 7)	81 (77–86)	33.83***	–	71 (66–75)	59.66***	–	2.79	0.27
	AD at follow-up (*n* = 7)	81 (76–85)	32.03***	–	70 (66–74)	51.90***	–	2.70	0.27
	Follow-up ≤24 months (*n* = 5)^a^	73 (65–80)	18.67**	6.76 (*p* = 0.009)	69 (63–75)	15.69**	1.61 (*p* = 0.20)	2.36	0.39
	Follow-up > 24 months (*n* = 5)	84 (78–88)	22.45***		74 (69–79)	42.89***		3.23	0.21
	Age ≤ 70 (*n* = 5)^a^	77 (71–82)	26.20***	5.77 (*p* = 0.02)	74 (69–79)	31.33***	7.29 (*p* = 0.007)	2.96	0.31
	Age > 70 (*n* = 4)	86 (80–91)	5.95		63 (56–70)	13.71**		2.32	0.22
	xMAP (*n* = 4)	88 (83–92)	6.04	20.18 (*p* < 0.001)	67 (61–73)	16.41***	3.30 (*p* = 0.07)	2.67	0.18
	ELISA (*n* = 6)^a^	70 (63–77)	21.66**		74 (69–79)	40.48***		2.69	0.41
T-tau	Global (*n* = 8)^a^	72 (66–77)	18.62*	–	70 (66–74)	60.06***	–	2.40	0.40
	Amnestic MCI (*n* = 5)	70 (64–77)	12.34*	–	68 (63–74)	30.68***	–	2.19	0.44
	AD at follow-up (*n* = 5)^a^	72 (66–78)	13.46*	–	65 (60–70)	18.45**	–	2.06	0.43
	Follow up ≤24 months (*n* = 5)^a^	73 (65–80)	11.94*	0.32 (*p* = 0.57)	75 (69–80)	43.58***	4.83 (*p* = 0.03)	2.92	0.36
	Follow up > 4 months (*n* = 3)	70 (61–78)	6.36*		65 (59–72)	11.65**		2.00	0.46
	Age ≤70 (*n* = 3)^a^	73 (64–80)	4.63	0 (*p* = 1)	69 (63–75)	31.98***	0.35 (*p* = 0.55)	2.36	0.39
	Age > 70 (*n* = 4)	73 (65–79)	10.58*		67 (60–73)	10.70*		1.97	0.40
	xMAP (*n* = 3)	69 (60–77)	4.45	0.97 (*p* = 0.32)	69 (62–75)	6.77*	0.21 (*p* = 0.65)	2.23	0.45
	ELISA (*n* = 5)^a^	74 (66–81)	13.20*		71 (65–76)	53.08***		2.55	0.37
p-tau	Global (*n* = 5)	63 (55–71)	21.22***	–	76 (70–80)	51.57***	–	2.63	0.49
	Amnestic MCI (*n* = 3)	56 (47–65)	8.66*	–	80 (74–85)	33.41***	–	2.80	0.55
	AD at follow-up (*n* = 3)	59 (51–68)	15.05***	–	71 (65–77)	35.49***	–	2.04	0.58
	Follow up ≤24 months (*n* = 2)	84 (64–95)	0.07	6.1 (*p* = 0.01)	93 (83–98)	1.92	14.16 (*p* < 0.001)	12.00	0.17
	Follow up > 24 months (*n* = 3)	59 (51–68)	15.05***		71 (65–77)	35.49***		2.03	0.58
	Age ≤ 70	–	–	–	–	–	–	–	–
	Age > 70 (*n* = 3)	59 (51–68)	13.79**		81 (75–86)	34.23***		3.10	0.50
	xMAP (*n* = 3)	57 (48–66)	11.01**	10.06 (*p* = 0.001)	78 (72–84)	25.48***	2.63 (*p* = 0.10)	2.59	0.55
	ELISA (*n* = 2)	85 (69–95)	0.15		69 (59–79)	23.46***		2.74	0.22
Aβ_42_/T-tau	Global (*n* = 5)^a^	86 (81–90)	21.20***	–	60 (54–65)	42.52***	–	2.15	0.23
	Amnestic MCI (*n* = 3)	89 (83–93)	10.62**	–	54 (48–61)	22.87***	–	1.94	0.20
	AD at follow-up (*n* = 4)^a^	86 (81–91)	21.20***	–	59 (54–64)	41.15***	–	2.10	0.24
	Follow up ≤ 24 months (*n* = 2)^a^	94 (87–98)	3.86	8.83 (*p* = 0.003)	48 (39–56)	9.00*	13.50 (*p* < 0.001)	1.81	0.13
	Follow up > 24 months (*n* = 3)	81 (73–87)	8.51*		67 (61–73)	20.02***		2.46	0.28
	Age ≤ 70 years (*n* = 2)^a^	88 (79–93)	18.63***	0.27 (*p* = 0.6)	56 (48–64)	29.63***	1.83 (*p* = 0.18)	2.00	0.21
	Age > 70 years (*n* = 3)	85 (78–91)	2.30		63 (56–70)	11.06**		2.30	0.24
	xMAP (*n* = 3)	85 (78–91)	2.30	0.27 (*p* = 0.6)	63 (56–70)	11.06**	1.83 (*p* = 0.18)	2.30	0.24
	ELISA (*n* = 2)^a^	88 (79–93)	18.63***		56 (48–64)	29.63***		2.00	0.21
Aβ_42_/p-tau	Global (*n* = 6)	85 (80–89)	12.08*	–	79 (74–83)	44.03***	–	4.05	0.19
	Amnestic MCI (*n* = 4)	88 (83–92)	2.70	–	78 (72–83)	41.01***	–	4.00	0.15
	AD at follow-up (*n* = 5)	85 (80–89)	12.08*	–	79 (74–84)	43.77***	–	4.05	0.19
	Follow up ≤ 24 months	–	–	–	–	–	–	–	–
	Follow up > 24 months (*n* = 5)	85 (80–89)	12.08*		79 (74–84)	43.77***		4.05	0.19
	Age ≤70 years (*n* = 3)	81 (73–88)	7.19*	3.59 (*p* = 0.06)	91 (86–95)	2.07	31.79 (*p* < 0.001)	9.00	0.21
	Age > 70 years (*n* = 3)	89 (83–94)	1.30		66 (58–74)	10.17**		2.62	0.17
	xMAP (*n* = 4)	89 (84–93)	1.47	7.83 (*p* = 0.005)	73 (67–79)	25.23***	16.76 (*p* < 0.001)	3.30	0.15
	ELISA (*n* = 2)	73 (59–84)	2.78^†^		91 (84–96)	2.04		8.11	0.30

^†^*p* < 0.10; *p < 0.05; **p < 0.01; ***p < 0.001.

*^a^Number of estimations is *n*+1 due to results were reported separately for amnestic MCI and non-amnestic MCI patients in Vos et al. (2013)*.

**Q*, heterogeneity (Cochran *Q*-test); LR+, positive likelihood ratio; LR−, negative likelihood ratio; Global, global meta-analysis; *n*, number of studies included in the meta-analysis; MCI, mild cognitive impairment; AD, Alzheimer’s disease*.

### Subgroups meta-analyses: Identification of clinical situations with increased CSF biomarkers’ predictive performance

Table 1 shows the list of potential confounding factors defined *a priori* based on the literature. Due to the low variability across the studies, it was only possible to perform subgroups meta-analyses for the following factors: (1) MCI subtype (studies exclusively including amnestic MCI cases); (2) diagnosis at follow-up (studies including MCI patients that converted exclusively to AD); (3) follow-up length (as rough estimation of time to AD conversion: ≤24 months vs. > 24 months); (4) age (studies with mean age younger vs. older than 70 years); and (5) technology for CSF analysis (ELISA vs. xMAP). As controlled factors, all the studies included samples from specialized centers; lacked postmortem AD confirmation; used comparable diagnostic criteria for MCI; applied NINCDS-ADRDA criteria for AD diagnosis [except one study (Herukka et al., 2005)]; and included MCI cases with similar global cognitive status (i.e., MMSE). Information on MCI severity according to MRI rating, gender distribution, level of education, family history of AD, and APOE e4 status was scarce or absent.

Table 3 shows sensitivity and specificity values with 95% CI, inter-groups difference (*Q*_BET_), heterogeneity, and likelihood ratios. Heterogeneity was significant in most of the subgroups meta-analyses. Noteworthy, CSF biomarkers’ predictive performance was optimal (>80%) in two clinically relevant situations, and heterogeneity was no longer significant: (1) P-tau alone had 84% sensitivity and 93% specificity for MCI cases converting to AD in ≤24 months, significantly different from 59% sensitivity (*p* = 0⋅01) and 71% specificity (*p* < 0⋅001) in studies with follow-up periods > 24 months; (2) Aβ_42_/p-tau ratio showed 81% sensitivity and 91% specificity in MCI patients younger than 70 years, significantly different from 66% specificity in MCI patients older than 70 years (*p* < 0⋅001).

Aβ_42_/p-tau ratio showed the best performance across the different subgroups meta-analyses. Sensitivity was slightly increased in studies including only amnestic MCI cases (heterogeneity no longer significant), MCI patients older than 70 years (heterogeneity no longer significant), and studies using ELISA. Aβ_42_/T-tau ratio yielded optimal sensitivity values, but suboptimal specificity. Results were not satisfactory for single CSF biomarkers, except for the remarkably good p-tau diagnostic performance commented above.

The analysis of positive likelihood ratios showed extremely high increase in the probability that the disease is present (LR+ = 12) for p-tau in MCI cases converting to AD in ≤24 months (Figure 2; Table 3). Moreover, there was a moderate increase in the probability that the disease is present (LR+ = 5–10) for the Aβ_42_/p-tau ratio in two situations: MCI patients younger than 70 years; and studies using ELISA technology. The analysis of negative likelihood ratios showed moderate decrease in the probability that the disease is present (LR– = 0.1–0.2) in several situations: p-tau and Aβ_42_/T-tau ratio in MCI cases converting to AD in ≤24 months; Aβ_42_/p-tau ratio in the global meta-analysis as well as in all the subgroups meta-analyses (except in MCI patients younger than 70 years and studies using ELISA technology).

## Discussion

The two main findings in this study are that the Aβ_42_/p-tau ratio has high capacity to predict AD conversion in MCI patients younger than 70 years; and p-tau alone has high capacity to identify MCI cases converting to AD in ≤24 months. The analysis of likelihood ratios showed that, in both situations, a CSF test result indicating pathological values of Aβ_42_/p-tau or p-tau significantly increase the probability that the disease is present [in this context progression from MCI to AD or, in other words, MCI due to AD (Albert et al., 2011)].

Better predictive performance of the CSF biomarkers in younger MCI patients has been recently shown in a large multi-center study (Mattsson et al., 2012). A fact that may explain this result is that typical AD brain alterations increase with age in individuals without dementia (Green et al., 2000; Bennett et al., 2006), with about a third of cognitively normal elderly evidencing an AD-like pattern of CSF biomarker alterations (Ewers et al., 2007; Bouwman et al., 2009; Mattsson et al., 2009; Shaw et al., 2009). This also occurs in stable MCI cases, therefore obstructing specificity for AD and undermining CSF biomarkers’ performance.

Regarding time to AD conversion, Gaser et al. (2013) showed that the CSF biomarkers had generally better performance for MCI cases that converted to AD in <12 months as compared with MCI cases that converted to AD in >12 months. In the current meta-analysis, this finding is still valid when considering 24 months as threshold. However, Buchhave et al. (2012) reported that the combination of CSF biomarkers might not be recommendable at 60 months before AD conversion. The reason for this is that at that point, many MCI-C have normal T-tau levels but already pathological Aβ_42_ levels. In another study, the combination of CSF biomarkers with structural MRI showed >80% sensitivity during the first 18 months of follow-up, decreasing to 75% at 24 months, and to 68% at 36 months (Westman et al., 2012). Therefore, predictive value and biomarkers’ utility strongly depend on the stage of the disease and time to conversion. Aβ_42_ performs better than Tau 5–10 years before conversion to AD, but T-tau and p-tau have better predictive power 0–5 years before conversion (Buchhave et al., 2012). Other biomarkers such as those based on structural MRI have the highest performance the closer to AD diagnosis. Future research should thus pursue in combining the CSF biomarkers not only with each other but also with other biomarkers. Recent studies show an increase in the diagnostic efficiency of CSF biomarkers when combined with neuroimaging biomarkers (Vos et al., 2012; Westman et al., 2012; Choo et al., 2013; Galluzzi et al., 2013; Prestia et al., 2013; Shaffer et al., 2013). The development of new combinations and indexes may contribute not only to predict AD conversion but, importantly, to facilitate prediction of time to conversion, which is still challenging.

Importantly, the Aβ_42_/p-tau ratio showed satisfactory predictive performance in a heterogeneous group of MCI patients, which better represents the clinical reality (global meta-analysis). Moreover, it is noteworthy that the sensitivity was increased in two specific conditions: amnestic MCI patients and old MCI patients (>70 years). Recently, Vos et al. (2013) showed that the CSF biomarkers are more sensitive in amnestic MCI than in non-amnestic MCI patients. An explanation for this is MCI heterogeneity. Only 30–60% of the MCI patients are affected by prodromal AD, whereas the others may stem from a variety of different etiologies and pathologies (Ritchie et al., 2001; Petersen, 2004). The amnestic subtype is mainly associated with AD pathology. Nonetheless, vascular etiology has also been referred as explicative factor, especially in those cases with cognitive impairment encompassing other domains besides memory (Petersen, 2004; Winblad et al., 2004). On the contrary, the non-amnestic subtype may have higher likelihood of progressing to non-AD dementias such as dementia with Lewy bodies or frontotemporal lobar degeneration (Petersen, 2004; Winblad et al., 2004). In this regard, it seems reasonable that the CSF biomarkers validated for AD perform better in the amnestic MCI cases. In agreement with Vos et al. (2013), this may have implications for clinical implementation of the new revised criteria for MCI (Albert et al., 2011), given that both amnestic and non-amnestic subtypes are considered in this criteria as possible prodromal stages of AD-type dementia. Regarding the finding of better CSF biomarkers’ sensitivity in old MCI patients, this is in line with the discussion above about the age-related increase in AD-like CSF biomarker patterns. Mattsson et al., 2012 also found increased sensitivity in MCI patients older tan 65 years compared to MCI patients younger than 65 years. On the other hand, Aβ_42_/p-tau specificity was not increased in any of the subgroups meta-analyses except for young MCI patients (≤70 years), as discussed above. The three CSF biomarkers alone and the Aβ_42_/T-tau ratio showed suboptimal predictive power except p-tau for MCI cases converting to AD in ≤24 months, as already commented.

A better performance of the Aβ_42_/p-tau ratio over the other CSF biomarkers has been reported in previous studies on MCI prediction (Hansson et al., 2006; Mattsson et al., 2009; Buchhave et al., 2012; Parnetti et al., 2012; Roe et al., 2013) and differential diagnosis between AD and other dementias (Maddalena et al., 2003; Jong et al., 2006; Holtzman, 2011). This finding is likely due to this ratio reflects two aspects of AD pathology, i.e., plaques (Aβ_42_), and neurodegeneration (tau). Moreover, p-tau usually shows better performance than T-tau (Mitchell, 2009; Bloudek et al., 2011; van Harten et al., 2011), probably because p-tau is not only a marker of axonal damage and neuronal degeneration, as T-tau, but it is more closely related to AD pathophysiology and the formation of neurofibrillary tangles (Anoop et al., 2010; Holtzman, 2011). In addition, CSF p-tau concentrations in dementia with Lewy bodies, frontotemporal lobar degeneration, and vascular dementia have been referred to be more comparable to concentrations in controls than to concentrations in AD patients (van Harten et al., 2011). This positively affects prediction of MCI due to AD.

Regarding the clinical value of the CSF biomarkers, results for negative likelihood ratios were normally better than results for positive likelihood ratios. This means that the CSF biomarkers are more useful to identify MCI patients that remain stable at follow-up (MCI-S) than to rule-in MCI patients that will progress to AD or dementia (MCI-C). This finding supports the consideration made in the new MCI diagnostic criteria in relation to biomarkers profile suggesting that the MCI syndrome is unlikely to be due to AD (point 3.6.4. in Albert et al. (2011): “the definitive absence of evidence of either Aβ deposition or neuronal injury strongly suggests that the MCI syndrome is not due to AD”). Our study shows that a normal result in the Aβ_42_/p-tau ratio has a moderate decrease in the probability that the disease is present (conversion to AD). This is true in all the situations evaluated in the different subgroups meta-analyses, although we could not confirm this for MCI cases converting to AD in ≤24 months because only one study was available (Monge-Argilés et al., 2011). This single study reported 86% sensitivity and 75% specificity (Monge-Argilés et al., 2011). Therefore, it is quite probable that a meta-analysis of the Aβ_42_/p-tau ratio in MCI cases converting to AD in ≤24 months would provide a satisfactory negative likelihood ratio, given that both p-tau and the Aβ_42_/T-tau ratio showed optimal results. On the other hand, positive likelihood ratios were normally within the range of a small increase in the probability that the disease is present. The only two situations where conclusive increase was achieved are those already commented above (Aβ_42_/p-tau ratio in young MCI patients and p-tau in MCI cases converting to AD in ≤24 months). This finding may have implications for the consideration made in the new MCI diagnostic criteria regarding biomarkers pattern indicating a high likelihood that the MCI syndrome is due to AD [*point 3.6.1*. in Albert et al., 2011]. In particular, young MCI patients with positive biomarkers of Aβ accumulation and neuronal injury seems to have increased risk to decline or progress to dementia due to AD in relatively short periods.

To determine in which specific situations the CSF biomarkers provide satisfactory predictive performance is of great relevance. In this study, some of those situations have been identified. However, despite these positive results, we acknowledge that much additional work needs to be done to validate the application of the CSF biomarkers as they are proposed in the new revised criteria for MCI (Albert et al., 2011). The main limitation for extending the use of the CSF biomarkers to the clinical routine is the difficulty to establish appropriate cut-offs. There is a big variability in the cut-offs applied across the different studies. This is in part related to differences in methodological aspects as well as absence of technical standardization. In this meta-analysis, two aspects related to variability in CSF methods were considered. First, we tried to analyze the influence of different cut-offs for the CSF biomarkers. Due to the great variability found it was not possible to group the studies in order to perform specific subgroups meta-analyses (Table 1). Second, the technology for the CSF analysis applied was also considered as potential confounding factor. Results showed that sensitivity and specificity values differed depending on whether xMAP (Luminex, Austin, US) or ELISA (Innogenetics, Ghent, Belgium) technology was used. A clear pattern was not found however. Therefore, future research is mandatory to hopefully ascertain universal cut-offs values for the CSF biomarkers. Several studies indicate that the standardization of laboratory procedures could contribute to reduce variability in the results (Hansson et al., 2006; Fagan et al., 2011; Mattsson et al., 2011).

Therefore, standardization of methodological aspects is expected to increase the clinical utility of the CSF biomarkers. In this meta-analysis, we demonstrate that several confounding factors are another source of variability in published diagnostic/predictive performance and cut-offs. We show that CSF biomarkers’ performance can be improved and heterogeneity reduced by carefully considering these confounding factors. In this regard, future studies should be addressed to explain how these factors influence the diagnostic and predictive performance of the CSF biomarkers. This need is reinforced by the fact that we could not evaluate 13 of the 18 identified potentially confounding factors given the lack of studies directly addressing these aspects. A related limitation is the scarce number of studies available for some of the analyses. This causes that certain subgroups meta-analyses could be influenced by some of the other confounding factors. In order to evaluate this, an analysis of coincident studies across factors was performed. Table S5 in Supplementary Material shows that most of the subgroups were rather independent from each other. However, for p-tau and Aβ_42_/p-tau, studies including follow-up periods >24 months coincided with studies with AD diagnosis at follow-up; and for p-tau and Aβ_42_/T-tau, studies using xMAP technology coincided with studies including MCI cases older than 70 years (and vice versa only for Aβ_42_/T-tau: ELISA technology with studies including MCI cases younger than 70 years). Another limitation is that systematic reviews and meta-analyses are essential tools for summarizing evidence accurately and reliably, but might be susceptible of bias if not properly conducted. Following PRISMA recommendations (Liberati et al., 2009; Moher et al., 2010), several strategies were carefully considered in this study to reduce risk of bias related to publication, data availability, and reviewer selection. Evidence was rigorously reviewed and literature was supplemented with manual query of relevant studies in order to minimize both publication and reviewer selection bias. Selected studies were carefully examined for clues suggesting that there may be missing results or data. Moreover, assessments were completed independently by more than one reviewer and consensus was required. Regarding the included studies, QUADAS-2 was applied to evaluate risk of bias and results applicability. It must be noticed that “domain 1” indicated high probability of patient selection bias in six of the included studies, related to inclusion of not completely consecutive or random samples, and not perfect avoidance of inappropriate exclusions. In particular, patients were normally selected from specialized centers on the basis of availability of CSF data, a procedure not generally performed in all incoming patients. This fact, may have certain impact in the applicability of the results, although these six studies scored rather well in the other three domains, indicating that the index test, the standard test, and flow and timing are not compromised. Another drawback is that the follow-up period was used as rough measure of time to AD conversion. Therefore, although it is clear that MCI-C cases in studies with follow-up ≤24 months converted to AD in less than 24 months, it is possible that some MCI-C cases in studies with follow-up >24 months also converted to AD before the threshold of 24 months. Finally, sensitivity and specificity values above 80% were considered indicative of optimal predictive performance according to international recommendations (The Ronald and Nancy Reagan Research Institute of the Alzheimer’s Association and the national Institute on Aging working Group, 1998). Higher levels are not easy to be achieved given that analyses are derived from clinically diagnosed AD cases in which the diagnostic accuracy already approximates 85% when validated by the standard pathologic diagnosis at autopsy (Mendez et al., 1992; Victoroff et al., 1995). None of the studies included in this meta-analysis performed postmortem AD confirmation. It is thus necessary to test CSF biomarkers’ predictive performance in pathologically confirmed AD patients.

In conclusion, this study contributes to define several situations in which the CSF biomarkers seem to be clinically useful for predicting conversion from MCI to AD. In particular, a baseline CSF test result indicating Aβ_42_/p-tau pathological values in MCI patients younger than 70 years has a moderate increase in the likelihood of developing AD. Moreover, a baseline CSF test result indicating pathological levels of p-tau increases the likelihood of developing AD within the next 24 months. To move forward in the knowledge about how different confounding factors influence the diagnostic and predictive performance of the CSF biomarkers is of utmost importance. Such knowledge will help the elaboration of a map of situations where the CSF biomarkers are useful, so that clinicians and researchers know when the new diagnostic criteria for MCI will be successful or otherwise prone to mistakes. This will be crucial when new disease-modifying treatments are available in the near future. Early prediction of MCI conversion to AD is expected to maximize treatment benefit if applied to the right people and before neuronal degeneration is too widespread and patients are already demented. In addition, this has ethical benefits because it is preferred not to treat patients with low risk of AD in trials that could cause side effects. Finally, this will also be important to enrich the samples with pure AD cases, both for research and clinical trials.

## Conflict of Interest Statement

The authors declare that the research was conducted in the absence of any commercial or financial relationships that could be construed as a potential conflict of interest.

## Supplementary Material

The Supplementary Material for this article can be found online at http://www.frontiersin.org/Journal/10.3389/fnagi.2014.00287/abstract
