# Supplementary material for: Improving CSF Biomarkers’ Performance for Predicting Progression from Mild Cognitive Impairment to Alzheimer’s Disease by Considering Different Confounding Factors: A Meta-Analysis
Source: Front Aging Neurosci. 2014 Oct 16;6:287. doi: 10.3389/fnagi.2014.00287 (PMC4199277; doi:10.3389/fnagi.2014.00287)
Supplement: Supplementary file 1 [file Data_Sheet1.PDF]

## SUPPLEMENTARY MATERIAL

## Supplementary Table 1: Search strategy for MEDLINE (OVID) database

| | |
|---|---|
| 1 | *Alzheimer Disease/cf, di [Cerebrospinal Fluid, Diagnosis] |
| 2 | *Cognition Disorders/cf, di, ge, ps [Cerebrospinal Fluid, Diagnosis, Genetics, Pathology] |
| 3 | 1 or 2 |
| 4 | *Amyloid beta-Peptides/cf, du [Cerebrospinal Fluid, Diagnostic Use] |
| 5 | *Biological Markers/an, cf [Analysis, Cerebrospinal Fluid] |
| 6 | *tau Proteins/cf [Cerebrospinal Fluid] |
| 7 | phosphorylated tau.mp. |
| 8 | total tau.mp. |
| 9 | T tau.mp. |
| 10 | P tau.mp. |
| 11 | A beta-42.mp. |
| 12 | *Cerebrospinal Fluid/an, di [Analysis, Diagnosis] |
| 13 | Cerebrospinal Fluid.mp. |
| 14 | CSF bioMarker.mp. |
| 15 | csf.mp. |
| 16 | Or/ 4 - 15 |
| 17 | 3 and 16 |
| 18 | limit 17 to "diagnosis (best balance of sensitivity and specificity)” |
| 19 | limit 18 to humans |

**Supplementary Table 2: Search strategy for EMBASE (Elsevier) database**

| | |
|---|---|
| 1 | 'alzheimer disease'/mj |
| 2 | 'cognitive defect'/mj |
| 3 | 1 OR 2 |
| 4 | 'amyloid beta protein'/mj |
| 5 | 'biological marker'/mj |
| 6 | 'tau protein'/mj |
| 7 | 'phosphorylated tau':ab,ti |
| 8 | 'total tau':ab,ti |
| 9 | 't tau':ab,ti |
| 10 | 'p tau':ab,ti |
| 11 | 'a beta-42':ab,ti |
| 12 | 'cerebrospinal fluid'/mj |
| 13 | 'cerebrospinal fluid':ab,ti |
| 14 | 'csf biomarker':ab,ti |
| 15 | csf:ab,ti |
| 16 | 4 OR 5 OR 6 OR 7 OR 8 OR 9 OR 10 OR 11 OR 12 OR 13 OR 14 OR 15 |
| 17 | 3 AND 16 |
| 18 | 'sensitivity and specificity'/exp |
| 19 | sensitivity:ab,ti |
| 20 | specificity:ab,ti |
| 21 | (('pre test' OR pretest) NEAR/8 probability):ab,ti |
| 22 | 'post-test probability':ab,ti |
| 23 | 'predictive value?':ab,ti |
| 24 | 'likelihood ratio?':ab,ti |
| 25 | 'diagnostic accuracy'/mj |
| 26 | 18 OR 19 OR 20 OR 21 OR 22 OR 23 OR 24 OR 25 |
| 27 | 17 AND 29 |

## Supplementary Table 3: Strategies followed to reduce the risk of bias

**Publication bias and reviewer selection bias**

1 *Systematic review:*
Evidence was rigorously reviewed in order to minimize both publication and reviewer selection bias.

2 *Manual query of relevant studies:*
Possible publication bias and reviewer selection bias was minimized by supplementing literature review with manual query of relevant studies.

3 *Examination of missing results or data:*
Selected studies were carefully examined for clues suggesting that there may be missing results or data.

**Data availability bias**

4 *Assessments were completed independently by more than one reviewer:*
Two reviewers (DF, AR) [1] independently sought for detailed data in all the identified studies. Peer review was done independently and in case of doubt and/or disagreements a third reviewer (LP) [2] was consulted.

**Methodological quality**

5 *QUADAS-2:*
Methodological quality was critically appraised with the QUADAS-2 scale. Assessment was performed in a blind manner by two reviewers (DF, AR) [1], independently, and in case of doubt and/or disagreements a third reviewer (LP) [2] was consulted.

6 *PRISMA statement for reporting systematic reviews with meta-analyses:*
The study was performed in accordance with the PRISMA statement, which provides a detailed guideline of a preferred reporting style of transparence.

[1] DF: Daniel Ferreira; AR: Amado Rivero-Santana; [2] LP: Lilisbeth Perestelo-Perez.

**Supplementary Table 4: Methodological quality of included studies (QUADAS-2): Risk of bias and applicability of the results**

| Authors (year) | DOMAIN 1 PATIENT SELECTION | | | | | DOMAIN 2 INDEX TEST | | | | DOMAIN 3 REFERENCE STANDARD | | | | DOMAIN 4 FLOW AND TIMING | | | |
|---|---|---|---|---|---|---|---|---|---|---|---|---|---|---|---|---|---|
| | A-2 | A-3 | A-4 | RB | AP | A-2 | A-3 | RB | AP | A-2 | A-3 | RB | AP | A-3 | A-4 | A-5 | RB |
| Buchhave et al. (2012) | Y | Y | Y | **L** | **H** | Y | Y | **L** | **H** | U | U | **U** | **U** | Y | Y | N | **U** |
| Eckerstrom et al. (2010) | Y | Y | U | **U** | **U** | U | N | **H** | **L** | U | U | **U** | **U** | U | Y | Y | **U** |
| Ewers et al. (2012) | N | Y | N | **H** | **L** | Y | U | **U** | **U** | U | U | **U** | **U** | Y | Y | N | **U** |
| Gaser (2013) | N | Y | N | **H** | **L** | Y | U | **U** | **U** | U | U | **U** | **U** | Y | Y | N | **U** |
| Hampel et al. (2004) | N | Y | N | **H** | **L** | U | N | **H** | **L** | U | Y | **U** | **U** | U | Y | Y | **U** |
| Hertze et al. (2010) | U | Y | Y | **U** | **U** | Y | Y | **L** | **H** | U | U | **U** | **U** | Y | Y | N | **U** |
| Herukka et al. (2005) | U | Y | U | **U** | **U** | Y | Y | **L** | **H** | U | Y | **U** | **U** | Y | Y | Y | **L** |
| Monge-Argilés et al. (2011) | U | Y | N | **H** | **L** | Y | Y | **L** | **H** | U | U | **U** | **U** | U | Y | Y | **U** |
| Parnetti et al. (2006) | Y | Y | Y | **L** | **H** | U | Y | **L** | **H** | U | U | **U** | **U** | U | Y | Y | **U** |
| Parnetti et al. (2012) | Y | Y | U | **U** | **U** | U | N | **U** | **L** | U | U | **U** | **U** | Y | Y | Y | **L** |
| Toledo (2013) | N | Y | N | **H** | **L** | Y | Y | **L** | **H** | U | U | **U** | **U** | Y | Y | N | **L** |
| Vos et al. (2013) | U | Y | N | **H** | **L** | U | Y | **L** | **H** | U | Y | **U** | **U** | U | Y | N | **U** |

Key: For each domain, risk of bias and applicability are qualitatively labeled (high/low/unclear) based on different answers (yes/no/unclear). Regarding domain 3 *reference standard*, all the studies received the label "unclear" in questions A-2, RB, and AP about the reference standard (i.e. clinical diagnosis), because the clinical diagnosis do not have 100% accuracy when compared to the golden standard (i.e. postmortem AD confirmation). Regarding domain 4 *flow and timing*, question A-3 about the appropriate time interval between the index test (i.e. CSF biomarker

at baseline) and reference standard (i.e. clinical diagnosis at follow-up) was labeled as “yes” if the follow-up period was longer than 2 years. On the contrary, the study was labeled as “unclear” if the follow-up period was shorter than 2 years, given that such short periods might underestimate the actual rate of conversion to AD.

Abbreviations: RB = risk of bias; AP = applicability of the results; Y = yes (criterion fulfilled); N = no (criterion not fulfilled); U = unclear (it is not clear that the criterion has been fulfilled); L = low risk of bias; H = high risk of bias.

**Supplementary Table 5: Analysis of coincident studies across the different subgroups meta-analyses**

| Biomarker | Meta-analysis | Buchhave et al. (2012) | Eckerström et al. (2010) | Hampel et al. (2004) | Hertze et al. (2010) | Herukka et al. (2005) | Monge-Argilés et al. (2011) | Parnetti et al. (2006) | Parnetti et al. (2012) | Vos et al. (2013) | ADNI studies | | |
|---|---|---|---|---|---|---|---|---|---|---|---|---|---|
| | | | | | | | | | | | Ewers et al. (2012) | Gaser et al. (2013) | Toledo et al. (2013) |
| **Aβ42** | Global (n=10)‡ | | | | | | | | | | | | |
| | Amnestic MCI (n=7) | | | | | | | | | | | | |
| | AD at follow-up (n=7) | | | | | | | | | | | | |
| | F-up ≤ 24 m. (n=5)‡ | | | | | | | | | | | | |
| | F-up > 24 m. (n=5) | | | | | | | | | | | | |
| | Age ≤ 70 (n=5)‡ | | | | | | | | | | | | |
| | Age > 70 (n=4) | | | | | | | | | | | | |
| | xMAP (n=4) | | | | | | | | | | | | |
| | ELISA (n=6)‡ | | | | | | | | | | | | |
| **T-tau** | Global (n=8)‡ | | | | | | | | | | | | |
| | Amnestic MCI (n=5) | | | | | | | | | | | | |
| | AD at follow-up (n=5)‡ | | | | | | | | | | | | |
| | F-up ≤ 24 m. (n=5)‡ | | | | | | | | | | | | |
| | F-up > 24 m. (n=3) | | | | | | | | | | | | |
| | Age ≤ 70 (n=3)‡ | | | | | | | | | | | | |
| | Age > 70 (n=4) | | | | | | | | | | | | |
| | xMAP (n=3) | | | | | | | | | | | | |
| | ELISA (n=5)‡ | | | | | | | | | | | | |
| **p-tau** | Global (n=5) | | | | | | | | | | | | |
| | Amnestic MCI (n=3) | | | | | | | | | | | | |
| | AD at follow-up (n=3) | | | | | | | | | | | | |
| | F-up ≤ 24 m. (n=2) | | | | | | | | | | | | |
| | F-up > 24 m. (n=3) | | | | | | | | | | | | |
| | Age ≤ 70 | - | - | - | - | - | - | - | - | - | - | - | - |
| | Age > 70 (n=3) | | | | | | | | | | | | |

| **Biomarker** | **Meta-analysis** | Buchhave et al. (2012) | Eckerström et al. (2010) | Hampel et al. (2004) | Hertze et al. (2010) | Herukka et al. (2005) | Monge-Argilés et al. (2011) | Parnetti et al. (2006) | Parnetti et al. (2012) | Vos et al. (2013) | ADNI studies | | |
|---|---|---|---|---|---|---|---|---|---|---|---|---|---|
| | | | | | | | | | | | Ewers et al. (2012) | Gaser et al. (2013) | Toledo et al. (2013) |
| | xMAP (n=3) | | | | | | | | | | | | |
| | ELISA (n=2) | | | | | | | | | | | | |
| **$A\beta_{42}$/ T-tau** | Global (n=5)‡ | | | | | | | | | | | | |
| | Amnestic MCI (n=3) | | | | | | | | | | | | |
| | AD at follow-up (n=4)‡ | | | | | | | | | | | | |
| | F-up ≤ 24 m. (n=2) | | | | | | | | | | | | |
| | F-up > 24 m. (n=3) | | | | | | | | | | | | |
| | Age ≤ 70 years (n=2)‡ | | | | | | | | | | | | |
| | Age > 70 years (n=3) | | | | | | | | | | | | |
| | xMAP (n=3) | | | | | | | | | | | | |
| | ELISA (n=2)‡ | | | | | | | | | | | | |
| **$A\beta_{42}$/ p-tau** | Global (n=6) | | | | | | | | | | | | |
| | Amnestic MCI (n=4) | | | | | | | | | | | | |
| | AD at follow-up (n=5) | | | | | | | | | | | | |
| | F-up ≤ 24 m. | - | - | - | - | - | - | - | - | - | - | - | - |
| | F-up > 24 m. (n=5) | | | | | | | | | | | | |
| | Age ≤ 70 years (n=3) | | | | | | | | | | | | |
| | Age > 70 years (n=3) | | | | | | | | | | | | |
| | xMAP (n=4) | | | | | | | | | | | | |
| | ELISA (n=2) | | | | | | | | | | | | |

Key: Coincident studies are represented in blue and orange. Within each biomarker (or ratio), cells with the same colour represent subgroups meta-analyses with coinciding studies (e.g. for p-tau, in orange, the meta-analysis for “AD at follow-up” includes the same three studies than the meta-analysis for “F-up > 24 m.”).

Abbreviations: Global = global meta-analysis; MCI = mild cognitive impairment; AD = Alzheimer’s disease; F-up = follow-up; m.: months; n = number of studies included in the meta-analysis; ‡ = number of estimations is n+1 due to results were reported separately for amnestic MCI and non-amnestic MCI patients in Vos et al. (2013).
